# LAMP‐Based Detection of *Candida albicans*: A Potential Tool for Candidemia Diagnosis

**DOI:** 10.1155/cjid/1980977

**Published:** 2026-06-19

**Authors:** Eakkapote Prompunt, Kannika Jamnai, Nawapol Marome, Nathaporn Phutthawong, Thitima Sumphanapai, Parin Kamseng, Chiraphat Kloypan, Somphot Saoin, Supaporn Khamchun, Sawitree Nangola

**Affiliations:** ^1^ Division of Clinical Microbiology and Medical Parasitology, Department of Medical Technology, School of Allied Health Sciences, University of Phayao, Mae Ka, Phayao, 56000, Thailand, up.ac.th; ^2^ Research Unit of Biomedical Molecular Innovation, University of Phayao, Mae Ka, Phayao, 56000, Thailand, up.ac.th; ^3^ Department of Medical Technology, School of Allied Health Sciences, University of Phayao, Mae Ka, Phayao, 56000, Thailand, up.ac.th; ^4^ Department of Chemistry, School of Sciences, University of Phayao, Mae Ka, Phayao, 56000, Thailand, up.ac.th; ^5^ Division of Clinical Hematology and Microscopy, Department of Medical Technology, School of Allied Health Sciences, University of Phayao, Mae Ka, Phayao, 56000, Thailand, up.ac.th; ^6^ Department of Pathology, School of Medicine, University of Phayao, Mae Ka, Phayao, 56000, Thailand, up.ac.th; ^7^ Division of Clinical Immunology and Transfusion Sciences, Department of Medical Technology, School of Allied Health Sciences, University of Phayao, Mae Ka, Phayao, 56000, Thailand, up.ac.th

**Keywords:** *Candida albicans*, candidemia, *ITS2*gene, LAMP technique

## Abstract

Candidemia is a life‐threatening bloodstream infection, most commonly caused by *Candida albicans* (*C. albicans*), and remains a significant concern in immunocompromised patients. Rapid and accurate species‐level detection is essential for effective clinical management. Loop‐mediated isothermal amplification (LAMP) is a robust nucleic acid amplification method that enables rapid pathogen detection under isothermal conditions. This study developed and evaluated an *ITS2*‐targeted LAMP assay for the detection of *C. albicans* in human blood. Analytical sensitivity and specificity were assessed using tenfold serial dilutions of DNA from a standard *C. albicans* strain (1 ng–1 fg). Specificity was tested against six other fungal species commonly involved in bloodstream infections: *C. tropicalis*, *C. krusei*, *C. neoformans*, *Fusarium* spp., *Aspergillus flavus*, and *A. niger*. The assay detected *C. albicans* DNA at concentrations as low as 10 fg, surpassing conventional PCR, which detected down to 10 pg. No cross‐reactivity was observed with non‐albicans species tested. Performance in blood was evaluated using artificially spiked samples, in which the assay consistently detected *C. albicans* at 10^1^ CFU/mL under controlled experimental conditions. These findings demonstrate that the *ITS2*‐based LAMP assay provides a rapid and sensitive molecular approach for the detection of *C. albicans* under the experimental conditions evaluated and may have potential as a complementary tool for candidemia detection following further clinical validation.

## 1. Introduction

*Candida albicans* (*C. albicans*) is a diploid fungus and a prominent human pathogen, commonly residing as a commensal organism in the gastrointestinal tract, oral cavity, and female genital tract. Under certain conditions, however, it can act as an opportunistic pathogen, causing infections that range from mild mucosal disorders, such as oral thrush and vaginal candidiasis, to life‐threatening systemic diseases, including candidemia. Among the approximately 150 species within the *Candida* genus, *C. albicans* remains the most clinically significant, underscoring its importance in both healthcare and research contexts [1].

Candidemia, a serious bloodstream infection predominantly caused by *Candida*, particularly *C. albicans*, is most frequently observed in immunocompromised individuals, such as patients undergoing chemotherapy or those with underlying immune deficiencies [2, 3]. Recognized as one of the most common types of nosocomial bloodstream infections, candidemia contributes substantially to patient morbidity and mortality, with rates reaching up to 60% in vulnerable populations [4, 5]. The recent rise in candidemia cases, coupled with its association with severe clinical outcomes, highlights the urgent need for timely and accurate diagnostic and therapeutic strategies. Early diagnosis, however, is challenging due to nonspecific clinical manifestations, including fever, chills, and fatigue, which often mimic other infectious conditions [6, 7].

Accurate detection and species‐level identification of *Candida* are therefore critical for effective prevention, control, and management of infections. In routine clinical laboratories, traditional blood cultures remain the gold standard, complemented by a variety of conventional diagnostic techniques. These include CHROMagar Candida for preliminary differentiation, germ tube formation for the rapid identification of *C. albicans*, chlamydoconidia production for morphological confirmation, and carbohydrate assimilation assays for species discrimination [8, 9]. Among these, CHROMagar Candida is one of the most widely used culture media, providing preliminary identification based on colony coloration. While rapid and cost‐effective, this approach has limited discriminatory power, as similar pigmentation among species can complicate accurate differentiation [10, 11]. Similarly, other conventional methods, though valuable, may fail to precisely identify uncommon or non‐albicans species. Accurate and timely differentiation between *C. albicans* and non‐albicans *Candida* is especially crucial in patients with recurrent infections [12]. Moreover, these methods are often time‐consuming, require skilled interpretation, and may yield ambiguous or false results, emphasizing the need for more reliable diagnostic strategies to enhance laboratory accuracy and support effective clinical management of candidiasis.

In this context, loop‐mediated isothermal amplification (LAMP) has emerged as a powerful nucleic acid amplification technique for the rapid and specific identification of pathogens under isothermal conditions [13, 14]. One of the key advantages of LAMP is its operation at a constant temperature, eliminating the need for a thermal cycler, which is required for conventional PCR. This allows the assay to be conducted using simple equipment, such as water baths, making it particularly suitable for resource‐limited settings [13, 14]. LAMP reactions are typically completed within 30–60 min, considerably faster than traditional PCR, which requires multiple thermal cycles and subsequent gel electrophoresis for visualization [13, 14]. The method demonstrates high specificity and sensitivity by employing four to six primers targeting multiple regions of the DNA, thereby enhancing amplification efficiency [13–15]. Furthermore, LAMP can be performed directly from infected tissues, reducing both preparation time and procedural complexity, and providing a rapid and reliable alternative for pathogen detection [13–15].

Despite the well‐recognized advantages of the LAMP technique for rapid and specific pathogen detection, its application for identifying *C. albicans* in cases of candidemia remains underexplored. This study therefore aims to thoroughly evaluate the performance of the LAMP assay for the direct detection of *C. albicans* from human blood samples. In particular, it seeks to assess the sensitivity, specificity, and overall diagnostic accuracy of LAMP in comparison with conventional polymerase chain reaction (PCR), providing essential insights into its potential as a rapid, reliable tool for timely diagnosis and improved clinical management of candidemia.

## 2. Materials and Methods

### 2.1. Fungal Strains and Culture Conditions

Clinical reference strains of *Candida albicans (C. albicans)* DMST 5815, *Candida tropicalis (C. tropicalis)* DMST 15495, *Candida krusei (C. krusei)* DMST 15317, *Cryptococcus neoformans (C. neoformans)* DMST 15319, *Fusarium* spp., *Aspergillus flavus (A. flavus)* DMST 22950, and *Aspergillus niger (A. niger)* DMST 15538 were kindly provided by the Division of Clinical Microbiology, Department of Medical Technology, Faculty of Allied Health Sciences, University of Phayao. All isolates were subcultured on Sabouraud dextrose agar (SDA) plates and incubated for 2–7 days under appropriate conditions to ensure optimal growth and purity. Identification of *Candida* species was carried out using conventional diagnostic methods, including CHROMagar™ Candida, germ tube formation, and chlamydoconidia production. For non‐*Candida* fungal species, identity and purity were verified using standard microbiological approaches, including colony morphology and microscopic examination (e.g., lactophenol cotton blue staining). In addition, all isolates were independently verified by experienced clinical microbiologists prior to subsequent experiments.

### 2.2. Fungal DNA Extraction

Genomic DNA was extracted from fungal isolates using a silica column–based purification method (Fungal DNA Extraction Kit [Vivantis Technologies, Selangor Darul Ehsan, Malaysia]) following the manufacturer’s protocol. Briefly, 1–3 mL of fungal cultures grown overnight or at logarithmic phase was harvested by centrifugation at 6000 × *g* for 5 min at room temperature, and the supernatant was completely discarded. The cell pellets were resuspended in 280 μL Buffer FuL and homogenized thoroughly by vortexing. Subsequently, 20 μL of proteinase K was added, and the samples were incubated at 65°C for 30 min with intermittent mixing to ensure complete lysis. The lysates were centrifuged at ≥ 14,000 × *g* for 5 min, and the supernatants were transferred into fresh tubes. Two volumes of Buffer FuB were added, followed by incubation at 65°C for 10 min. DNA was precipitated by the addition of 200 μL absolute ethanol and immediately mixed to avoid uneven nucleic acid precipitation. The mixtures were then applied to silica spin columns and centrifuged at 10,000 × *g* for 1 min. Columns were washed twice with 650 μL of wash buffer, followed by centrifugation to remove residual ethanol. Finally, DNA was eluted in 50 μL of preheated elution buffer and stored at −20°C until use. DNA concentration and purity were measured using a NanoDrop spectrophotometer (Thermo Fisher Scientific, Madison, USA).

### 2.3. Optimization of LAMP Reaction

Five oligonucleotide primers recognizing seven conserved regions within the internal transcribed spacer 2 (*ITS2*) gene of *C. albicans* were employed for the LAMP assay (Table 1), as previously described by Kasahara et al. These primers consisted of F3 (Forward Outer Primer) and B3 (Backward Outer Primer), FIP (Forward Inner Primer), and BIP (Backward Inner Primer), and a loop primer (LB), which together facilitate strand displacement amplification and enhance reaction efficiency [16]. The LAMP reaction was performed in a final volume of 25 μL containing 1× isothermal buffer (New England Biolabs Inc., Ipswich, MA, USA), 6 mM MgSO_4_ (New England Biolabs Inc.), 1.4 mM dNTPs (Vivantis Technologies, Malaysia), 1.4 mM dUTP (New England Biolabs Inc.), 1.6 μM each of FIP and BIP, 0.2 μM each of F3 and B3, 0.4 μM of LB, 0.32 U Bst DNA polymerase (New England Biolabs Inc.), 0.005 U Antarctic Thermolabile Uracil DNA Glycosylase (UDG) (New England Biolabs Inc.), 0.5 μL of 50X Fluorescent dye (New England Biolabs Inc., Ipswich, MA, USA), and DNA template from fungal species. To minimize the risk of carryover contamination, all experimental procedures were conducted under strict contamination control conditions. Preamplification (reagent preparation and DNA extraction) and postamplification (product analysis) steps were performed in physically separated areas with dedicated equipment and consumables. A unidirectional workflow was strictly maintained throughout the process. Aerosol‐resistant filter tips were used for all pipetting steps. In addition, dUTP was incorporated into the LAMP reaction mixture in combination with thermolabile UDG to prevent carryover contamination from previously amplified products. Primer sequences employed in this study are listed in Table 1.

**TABLE 1:** Nucleotide sequences of primers targeting the *ITS2* gene of *C. albicans* used in the LAMP assay.

Primer name	Sequence (5′–3′)	Length (nucleotides)	Target region	Reference
F3 (Forward Outer Primer)	TCT GGT ATT CCG GAG GGC	18	*ITS2*	Kasahara et al. [16]
B3 (Backward Outer Primer)	AGT CCT ACC TGA TTT GAG GT	20	*ITS2*	Kasahara et al. [16]
FIP (Forward Inner Primer)	CTA CCG TCT TTC AAG CAA ACC CAT GAG CGT CGT TTC TCCCT	41	*ITS2*	Kasahara et al. [16]
BIP (Backward Inner Primer)	TTG ACA ATG GCT TAG GTC TAA CCA AAA GAT ATA CGT GGT GGA CGT TAC	48	*ITS2*	Kasahara et al. [16]
LB (Loop Backward Primer)	CTC AAC ACC AAA CCC AGC GG	20	*ITS2*	Kasahara et al. [16]

The assay was optimized by testing five incubation temperatures (55°C, 60°C, 63°C, 65°C, and 70°C) and five reaction times (30 min, 40 min, 50 min, 60 min, and 70 min) to determine the most suitable conditions for amplification. Amplicons were first inspected by direct visual observation at the completion of each reaction. To further validate fluorescence‐based detection, all LAMP products were subjected to electrophoresis on 1.5% agarose gels and visualized under UV illumination. The presence of a characteristic ladder‐like banding pattern confirmed the successful amplification of target DNA by the designed primers.

### 2.4. Conventional PCR

A conventional PCR assay targeting the *ITS2* gene was performed to compare its sensitivity with that of the LAMP method. The F3 and B3 primers were used as forward and reverse primers, respectively. PCR amplification was carried out in a 20‐μL reaction mixture containing 2× Phusion Master Mix (Thermo Fisher Scientific, Madison, USA), 0.5 μM of each primer, DNA template ranging from 1 fg to 50 ng, and 0.6 μL dimethyl sulfoxide (DMSO). The cycling conditions consisted of an initial denaturation at 98°C for 30 s, followed by 30 cycles of 98°C for 10 s, 53°C for 30 s, and 72°C for 20 s, with a final extension at 72°C for 5 min. PCR products were stained with Novel Juice (Thermo Fisher Scientific), separated on 1.5% agarose gels, and visualized under UV illumination. A DNA ladder was included in each run to confirm the expected product size. Positive and negative controls were included to ensure assay validity. The expected size of the *ITS2* amplicon generated using the F3 and B3 primers was 214 bp.

### 2.5. Analytical Sensitivity of the LAMP Assay

The analytical sensitivity of the LAMP assay was evaluated using tenfold serial dilutions of genomic DNA extracted from *C*. *albicans*, ranging from 1 ng to 1 fg per reaction. This concentration range was selected to cover a broad dynamic range for sensitivity assessment and was based on the reported copy number of the *ITS2* gene in *C. albicans* (approximately 55–110 copies per cell), thereby reflecting biologically relevant gene copy numbers. Each dilution was tested in five independent replicates to determine the lowest DNA concentration that could be reliably detected. Serial dilutions were prepared in sterile nuclease‐free water and thoroughly mixed to ensure homogeneity. LAMP reactions were performed under the optimized conditions described above. Amplification was assessed by both visual inspection and agarose gel electrophoresis. Detection performance at each concentration was expressed as the proportion of positive results among the total number of replicates. Ninety‐five percent confidence intervals (95% CI) were calculated using the binomial exact (Clopper–Pearson) method. This approach allowed the determination of the detection limit of the LAMP assay, providing a benchmark for its analytical sensitivity and comparability with conventional PCR.

### 2.6. Analytical Specificity of the LAMP Assay

The analytical specificity of the LAMP assay for *C*. *albicans* detection was evaluated using genomic DNA from *C. albicans* and six additional fungal species commonly associated with bloodstream infections, including *C. tropicalis*, *C. krusei*, *C*. *neoformans*, *Fusarium* spp., *A*. *flavus*, and *A*. *niger*. Each DNA sample was tested in five independent replicates under the optimized LAMP conditions described above. Amplification was assessed by both direct visual inspection and agarose gel electrophoresis (1.5%), allowing verification of specificity through the presence or absence of the characteristic ladder‐like banding pattern. No amplification was observed for non‐albicans fungal species, whereas *C. albicans* DNA consistently produced a positive LAMP signal, indicating high analytical specificity without cross‐reactivity. Detection outcomes were expressed as the proportion of positive results among total replicates, and 95% CIs were calculated using the binomial exact (Clopper–Pearson) method. These results demonstrate that the LAMP assay exhibits high specificity and is suitable for the accurate detection of *C. albicans*.

### 2.7. Validation of the LAMP Assay for the Detection of *C. albicans* in Human Blood

Human blood samples were kindly provided by the Blood Bank, Department of Medical Technology, Phayao Hospital. All procedures involving human specimens were conducted in accordance with ethical guidelines and were approved by the Human Research Ethics Committee of the University of Phayao on Health Sciences and Sciences and Technology (HREC‐UP‐HSST 1.1/055/68).

To evaluate assay performance, *C*. *albicans* was cultured in 10 mL of Sabouraud dextrose broth (SDB) at 37°C for 24 h. Cell density was determined using a UV–Vis spectrophotometer at 600 nm (OD_600_ = 0.2), corresponding to the logarithmic growth phase. For sensitivity assessment, *C. albicans* cells were enumerated using a hemocytometer, and the cell concentration was adjusted accordingly. This suspension was subsequently spiked into human blood to obtain a final concentration of 1 × 10^6^ CFU/mL and used as a stock for serial dilutions. Aliquots (1 mL) of whole blood from five independent healthy donors were then spiked with *C. albicans* to achieve final concentrations ranging from 10^1^ to 10^6^ CFU/mL. Each sample was processed immediately for downstream analysis.

DNA was extracted from the spiked samples as described above and subjected to both the LAMP assay and conventional PCR. Amplification was initially assessed by direct visual inspection and subsequently confirmed by agarose gel electrophoresis (1.5%). The presence of a characteristic ladder‐like banding pattern was considered indicative of successful amplification. Detection outcomes were expressed as the proportion of positive results among the total number of donors, and 95% CI were calculated using the binomial exact (Clopper–Pearson) method.

Analytical specificity in blood samples was evaluated using *C. albicans* (10^6^ CFU/mL) and six additional fungal species commonly associated with bloodstream infections (10^6^ CFU/mL), including *C. tropicalis*, *C. krusei*, *C*. *neoformans*, *Fusarium* spp., *A*. *flavus*, and *A*. *niger*. DNA extracted from each sample was tested under the optimized LAMP conditions described above. Amplification results were assessed by visual inspection and confirmed by agarose gel electrophoresis. Detection outcomes were expressed as the proportion of positive results among total donors, and 95% CI were calculated using the binomial exact (Clopper–Pearson) method.

## 3. Results

### 3.1. Optimization of LAMP Reaction

**FIGURE 1:**
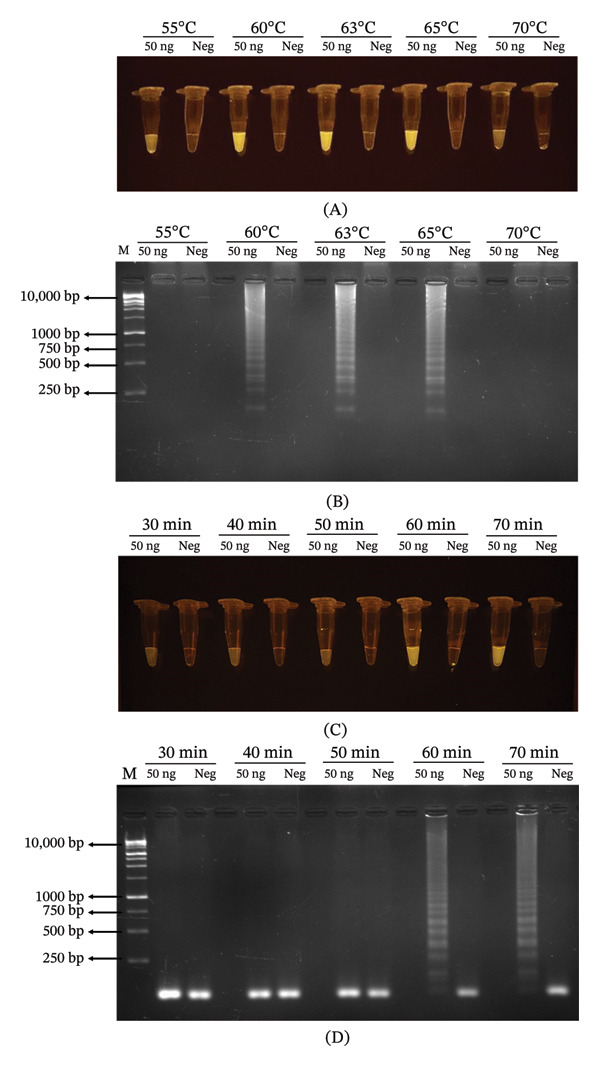
The optimum temperature and incubation time of the LAMP reaction. Five different temperatures, including 55°C, 60°C, 63°C, 65°C, and 70°C, at 1 h of incubation time (A, B). Five different reaction times, including 30 min, 40 min, 50 min, 60 min, and 70 min, at 60°C (C,D). Sample amount: 50 ng. M: 1 kb marker. Neg: sterile distilled water (negative control). LAMP, loop‐mediated isothermal amplification.

Five oligonucleotide primers designed to recognize seven conserved regions of the *ITS2* gene in *C. albicans* were employed in the LAMP assay [16]. The outcome of the LAMP reaction could be readily distinguished by the addition of LAMP fluorescent dye, whereby negative reactions retained a colorless or pale green, whereas positive reactions exhibited a distinct green fluorescence. To identify the optimal reaction temperature, LAMP assays were conducted for 1 h at five temperature conditions (55°C, 60°C, 63°C, 65°C, and 70°C), employing 50 ng of DNA extracted from *C. albicans* as templates. Positive amplification was obtained at 60°C, 63°C, and 65°C, whereas no detectable fluorescence signal was observed at 55°C and 70°C (Figure 1A). In addition, the positive LAMP reactions generated a characteristic ladder‐like pattern, consisting of multiple DNA fragments of varying sizes extending up to the loading well, when analyzed on a 1.5% agarose gel (Figure 1B). Among the effective temperatures, 60°C was selected for subsequent experiments as it consistently produced clear amplification signals and was considered optimal for stable and efficient reaction performance. Accordingly, 60°C was chosen as the reaction temperature for subsequent optimization.

Analysis of LAMP products obtained from varying incubation periods revealed the absence of green fluorescence and DNA laddering at 30 min, 40 min, and 50 min (Figure 1C). Conversely, clear positive signals accompanied by characteristic ladder‐like banding of comparable intensity were evident at 60 min and 70 min (Figure 1D). These results indicate that an incubation period of 60 min is sufficient to ensure completion of the LAMP reaction.

### 3.2. LAMP Reaction Specificity

To evaluate the specificity of the LAMP assay, genomic DNA extracted from *C. albicans* and six additional fungal species commonly associated with bloodstream infections, including *C. tropicalis, C. krusei, C. neoformans, Fusarium* spp., *A. flavus,* and *A. niger*, was subjected to analysis.

**FIGURE 2:**
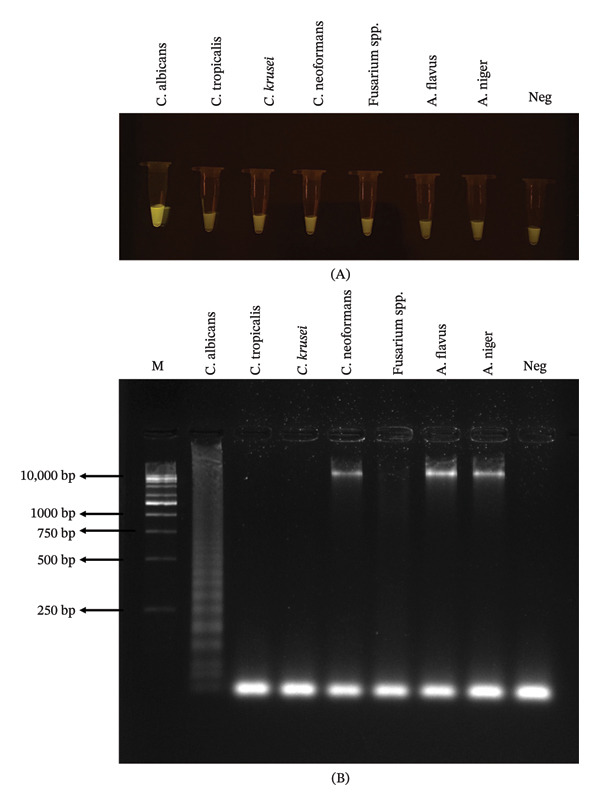
The specificity of the LAMP reaction. 50 ng of DNA from different fungal strains, including *C. albicans, C. tropicalis, C. krusei, C. neoformans, Fusarium* spp., *A. flavus,* and *A. niger*. M: 1 kb marker. Neg: sterile distilled water (negative control). LAMP, loop‐mediated isothermal amplification.

The assay produced a positive reaction exclusively for *C. albicans* (5/5 replicates (100%, 95% CI: 47.8%–100%) (Figure 2A), indicating high analytical specificity under the conditions tested. To further validate the fluorescence‐based detection, all LAMP products were analyzed using electrophoresis on a 1.5% agarose gel (Figure 2B). This analysis revealed the characteristic ladder‐like pattern in samples containing *C. albicans*. In contrast, no amplification or ladder‐like banding was observed in DNA samples derived from any of the other fungal species tested. The results indicated that the LAMP assay is highly selective for *C. albicans*, showing no cross‐reactivity with the other fungal species tested. The observed specificity and characteristic amplification pattern support the potential utility of the assay as a rapid molecular detection method for *C. albicans*, although further evaluation using a broader range of clinically relevant pathogens and clinical specimens will be necessary to confirm its diagnostic applicability.

### 3.3. Evaluation of the Sensitivity of the LAMP Assay in Comparison With Conventional PCR

**FIGURE 3:**
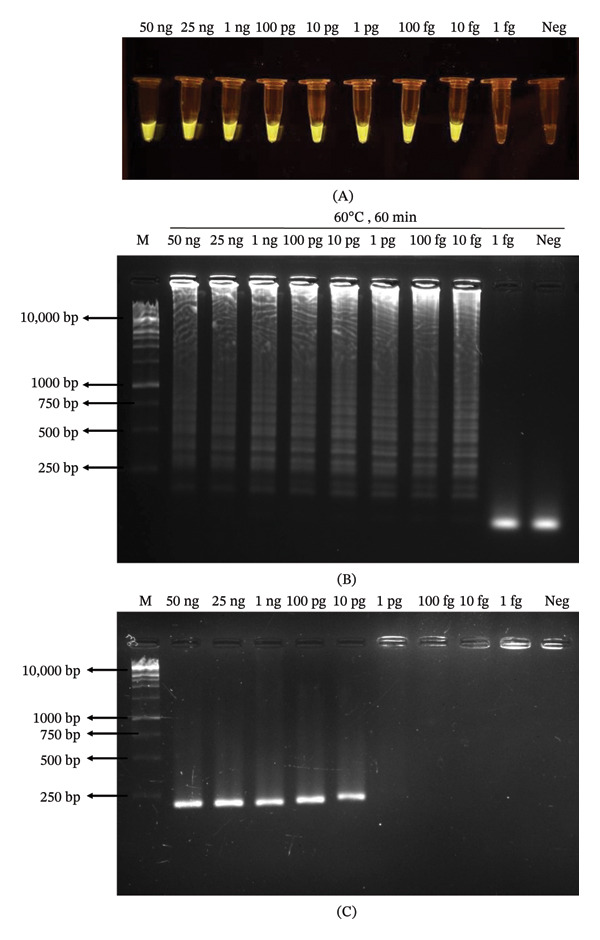
The sensitivity of the LAMP reaction. *C. albicans* DNAs were amplified by LAMP reaction at 60°C for 60 min (A) compared with the standard PCR (C). The gel electrophoresis images of LAMP‐amplified *C. albicans* DNA samples (B). M: 1 kb marker. Neg: sterile distilled water (negative control). LAMP, loop‐mediated isothermal amplification.

The analytical sensitivity of the LAMP assay was systematically evaluated using tenfold serial dilutions of DNA extracted from *C. albicans*, ranging from 50 ng to 1 fg, and was compared with a conventional PCR assay targeting the same *ITS2* region as a baseline analytical reference. Remarkably, the assay was able to detect DNA at concentrations as low as 10 fg, with positive amplification observed in 5/5 replicates (100%, 95% CI: 47.8%–100%) (Figure 3A), indicating the approximate analytical limit of detection. In contrast, conventional PCR yielded positive signals only down to 10 pg, also in 5/5 replicates (100%, 95% CI: 47.8%–100%) (Figure 3C). These findings indicate that the LAMP assay demonstrated greater analytical sensitivity than conventional PCR in this experimental setting. To validate the positive fluorescence observed in the reaction tubes, all LAMP products were subjected to electrophoresis on a 1.5% agarose gel (Figure 3B). The analysis revealed the characteristic ladder‐like pattern consistent with the fluorescence‐based detection results. These observations collectively confirm that the developed LAMP assay not only provides superior sensitivity compared with standard PCR but also ensures reliable amplification across a wide range of DNA concentrations. Collectively, these results demonstrated that the developed LAMP assay was capable of detecting low concentrations of *C. albicans* DNA, supporting the potential utility of the assay as a rapid molecular detection approach for *C. albicans*. However, the conventional PCR assay used in this study served primarily as a baseline analytical comparator and should not be interpreted as representing current standardized molecular diagnostic methods for candidemia.

### 3.4. Evaluation of the Efficiency of the LAMP Assay for Detecting *C. albicans* in Blood Samples

The performance of the LAMP assay for detecting *C*. *albicans* in blood samples was evaluated using blood from five healthy donors spiked with defined concentrations of the organism, ranging from 10^1^ to 10^6^ CFU/mL. This experimental design allowed for a systematic evaluation of the assay detection capacity under conditions that closely mimic clinically relevant scenarios. The performance of the LAMP assay was subsequently compared with that of a conventional PCR assay used as a baseline analytical comparator targeting the same *ITS2* region.

**FIGURE 4:**
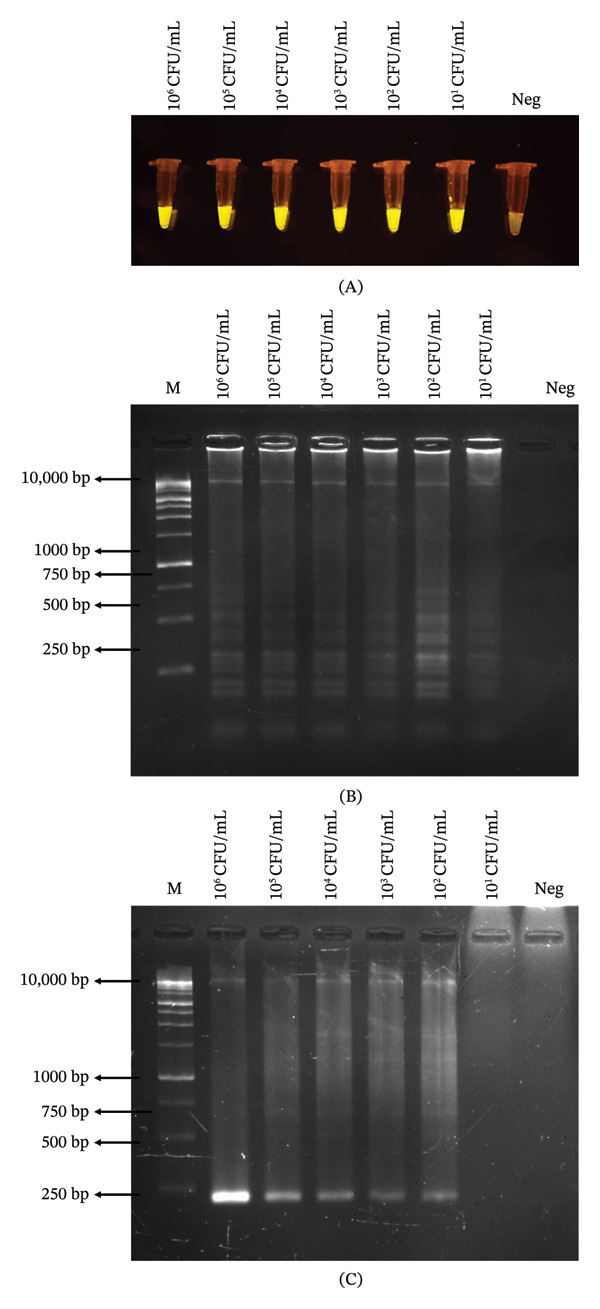
Detection of *C. albicans* in spiked human blood samples. *C. albicans* DNAs were amplified by LAMP reaction at 60°C for 60 min (A) compared with the standard PCR (C). The gel electrophoresis images of LAMP‐amplified *C. albicans* DNA samples (B). Samples: Human blood samples spiked with *C. albicans* from 10^1^ to 10^6^ CFU/mL, M: marker, Neg: sterile distilled water (negative control). LAMP, loop‐mediated isothermal amplification.

Notably, the LAMP assay consistently detected pathogen DNA at concentrations as low as 10^1^ CFU/mL across all five donor samples (5/5 donors, 100%, 95% CI: 47.8%–100%). In contrast, conventional PCR generated positive signals only at concentrations of 10^2^ CFU/mL, also across all five donor samples (5/5 donors, 100%, 95% CI: 47.8%–100%) (Figure 4A, C)). Under these test conditions, the LAMP assay was approximately one order of magnitude more sensitive than the comparison method. To further confirm the fluorescence‐based detection results, LAMP amplification products were analyzed by agarose gel electrophoresis. The characteristic ladder‐like banding pattern observed was consistent with successful LAMP amplification (Figure 4B). Collectively, these findings demonstrate that this assay was capable of detecting low concentrations of *C. albicans* in artificially spiked blood samples under controlled experimental conditions and support its potential utility as a rapid molecular detection approach. However, because the conventional PCR assay served primarily as a baseline analytical comparator rather than a clinically standardized molecular diagnostic method, and because evaluation was performed using spiked rather than clinical specimens, further validation using clinical samples and standardized molecular assays will be necessary to establish diagnostic applicability in bloodstream infections.

### 3.5. Evaluation of the Specificity of the LAMP Assay for Detecting *C. albicans* in Blood Samples

**FIGURE 5:**
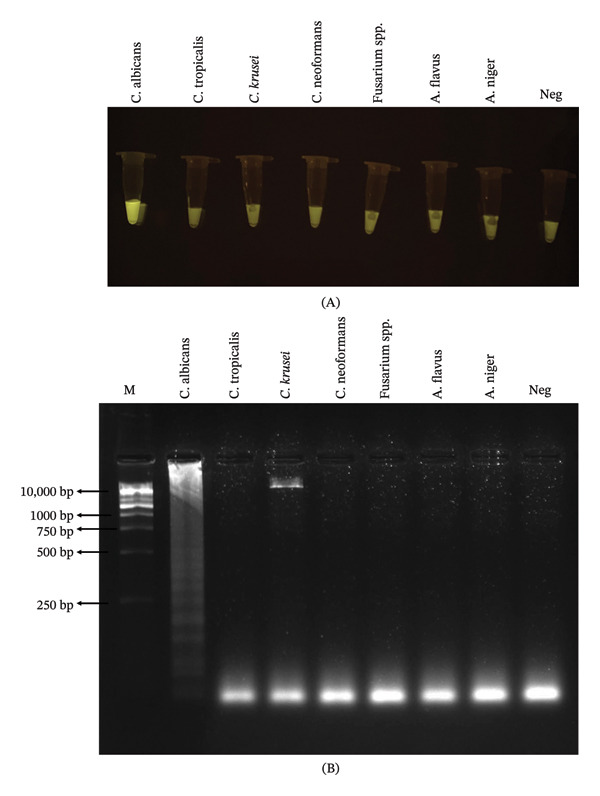
The specificity of the LAMP reaction for detecting *C. albicans* in blood samples. The visualized fluorescent and gel electrophoresis images of *C. albicans, C. tropicalis, C. krusei, C. neoformans, Fusarium* spp., *A. flavus,* and *A. niger*. Samples: human blood samples spiked with *C. albicans, C. glabrata, C. krusei, C. neoformans, Fusarium* spp., *A. flavus,* and *A. niger* at 10^6^ CFU/mL, M: 1 kb marker. Neg: sterile distilled water (negative control). LAMP, loop‐mediated isothermal amplification.

The specificity of the LAMP assay for detecting *C. albicans* in blood samples was evaluated by spiking human blood with a panel of fungi commonly associated with bloodstream infections, including *C. tropicalis*, *C. krusei*, *C*. *neoformans*, *Fusarium* spp., *A*. *flavus*, and *A*. *niger*, at a concentration of 10^6^ CFU/mL. The assay generated positive amplification exclusively in samples containing *C. albicans* across all five donor samples (5/5 donors, 100%, 95% CI: 47.8%–100%), while no amplification was observed for any of the other fungal species tested (Figure 5A). To further confirm the fluorescence‐based detection results, all LAMP products were analyzed by electrophoresis on a 1.5% agarose gel. The characteristic ladder‐like banding pattern, consisting of multiple DNA fragments of varying sizes, was observed only in samples containing *C. albicans* (Figure 5B). These findings indicated high analytical specificity of the LAMP assay for detecting *C. albicans* in blood samples under the experimental conditions evaluated. Nevertheless, further evaluation using a broader range of clinically relevant pathogens and clinical specimens will be necessary to more comprehensively assess its diagnostic applicability.

## 4. Discussion

The *ITS2* region of *C. albicans*, a clinically important pathogenic yeast, plays a pivotal role in ribosomal RNA (rRNA) maturation and is widely recognized as a reliable genetic marker in molecular and phylogenetic studies. As part of the ribosomal DNA (rDNA) repeat unit, *ITS2* exhibits an intriguing balance between structural conservation and sequence variability. This duality not only reflects its essential function in ribosome biogenesis but also provides a valuable molecular signature that distinguishes *C. albicans* from closely related *Candida* species. Consequently, *ITS2* has emerged as a robust tool for species identification, taxonomic classification, and the elucidation of evolutionary relationships within the genus [16, 17].

In clinical microbiology, the *ITS2* sequence has been extensively employed to achieve rapid and accurate species‐level identification, which is critical for guiding appropriate antifungal therapy. Given the rising incidence of invasive candidiasis and the intrinsic differences in antifungal susceptibility among *Candida* species, the discriminatory power of *ITS2* provides a significant advantage over conventional phenotypic methods. Furthermore, its sequence variability enables precise differentiation between closely related taxa, thereby reducing misidentification that often complicates clinical outcomes [16, 18].

A set of LAMP primers (F3, B3, FIP, BIP, and LB) was specifically designed to target seven conserved regions within the *ITS2* gene of *C*. *albicans* [16, 18]. In this study, the optimal conditions for the LAMP assay were identified as 60°C for 60 min, which successfully facilitated the amplification of DNA from the standard *C. albicans* strain. These parameters fall within the temperature range commonly reported for LAMP reactions, generally between 60°C and 65°C, where *Bst* DNA polymerase exhibits maximal activity and stability [13, 14]. Comparable findings have been documented in previous studies. Notomi et al. [19] first demonstrated efficient amplification at 63°C, while subsequent investigations on fungal pathogens, including *C. albicans*, typically reported optimal performance within the 60°C–65°C range with incubation times of approximately 1 h [18]. Likewise, Tomita et al. [20] suggested that a 60‐min incubation period is sufficient to achieve high sensitivity while minimizing nonspecific amplification. Taken together, the present findings are consistent with earlier reports, further supporting the robustness and reproducibility of the LAMP method under these conditions.

Fungal pathogens are increasingly recognized as significant causative agents of sepsis, particularly among critically ill or immunocompromised patients. Among these, *Candida* species represent the most frequent cause, with *C. albicans* being predominant, although non‐albicans species such as *C. glabrata, C. parapsilosis,* and *C. tropicalis* are rising in prevalence in healthcare settings [21, 22]. Other fungi, including *Aspergillus* spp., *Cryptococcus* spp., and *Fusarium* spp., can also cause systemic infections and sepsis, especially in patients with severe immunodeficiency [23, 24]. Risk factors for fungal sepsis include prolonged intensive care unit stays, central venous catheterization, broad‐spectrum antibiotic use, total parenteral nutrition, neutropenia, and recent surgery [21, 25]. Rapid and accurate detection of *C. albicans* and other fungal pathogens is therefore crucial for guiding antifungal therapy and improving patient outcomes. The *ITS2*‐targeted LAMP assay optimized in the present study provides a rapid and specific molecular detection approach under experimental conditions and may be useful for future diagnostic applications following further clinical validation.

LAMP is widely recognized for its high sensitivity in detecting fungal genes, surpassing traditional methods such as multiplex polymerase chain reaction (M‐PCR). Previous studies have reported that LAMP can achieve a sensitivity of 88%, compared to 56% for M‐PCR, highlighting its superior capability to detect low‐abundance targets within fungal DNA samples [26, 27]. Furthermore, LAMP has consistently outperformed conventional PCR techniques, successfully amplifying DNA at concentrations often too low for detection by PCR [27]. The quantitative performance of LAMP can be evaluated by performing serial dilutions of target DNA, thereby determining the minimal detectable amounts [28]. Collectively, these characteristics position LAMP as a robust and versatile tool, not only for the rapid and sensitive diagnosis of fungal infections but also for functional studies on fungal genetics, pathogenicity, and resistance mechanisms [28]. In the present study, the assay demonstrated remarkable sensitivity, detecting DNA at concentrations as low as 10 fg, whereas conventional PCR yielded positive signals only down to 10 pg, thereby confirming the significantly enhanced sensitivity of the LAMP method.

In the present study, the performance of the LAMP assay for detecting *C*. *albicans* in blood samples was systematically evaluated using spiked human blood with defined concentrations of the organism ranging from 10^1^ to 10^6^ CFU/mL. This experimental design enabled a controlled assessment of assay performance under conditions intended to approximate clinically relevant scenarios. Conventional PCR was included primarily as a baseline analytical comparator to facilitate relative assessment of amplification sensitivity using the same ITS2 target region, rather than as a representation of current clinical molecular diagnostic standards. In contemporary clinical practice, standardized qPCR and other validated molecular assays are generally preferred for candidemia detection because of their superior sensitivity, reproducibility, and quantitative capability. The LAMP assay consistently detected *C. albicans* at concentrations as low as 10^1^ CFU/mL, whereas conventional PCR yielded positive results only at 10^2^ CFU/mL, suggesting an approximately one‐log improvement in analytical sensitivity under the tested conditions. These findings are consistent with the known advantages of LAMP, including high amplification efficiency and tolerance to inhibitory substances commonly present in complex biological samples.

However, quantitative PCR (qPCR) is widely recognized as a reference molecular method for the detection of *C. albicans*, offering high analytical sensitivity—often reported to detect as low as 5–10 CFU/mL in blood samples—along with the advantage of quantitative output through cycle threshold (Ct) values [29, 30]. A limitation of this study is the absence of a direct comparison with qPCR. While conventional PCR was selected as a baseline method due to its accessibility and widespread use in many laboratory settings, future studies incorporating standardized qPCR assays will be necessary to provide a more comprehensive evaluation of diagnostic performance. However, the result of this study showed the same range of detection limit as shown in previous study [29].

Another important limitation is that assay performance in blood was evaluated primarily using artificially spiked samples rather than clinical specimens from patients with confirmed candidemia. Although spiked blood models are commonly used in early‐stage assay development to enable controlled evaluation of analytical sensitivity, they do not fully replicate the complexity of clinical samples. In real‐world settings, factors such as low fungal burden, intermittent fungemia, the presence of host‐derived inhibitors, and variability in sample processing can significantly impact diagnostic performance [30, 31]. Previous studies have shown that fungal loads in candidemia are often low, frequently below 1–10 CFU/mL, and that whole blood may contain substances that inhibit nucleic acid amplification [29, 30]. Additionally, this major limitation was unable to be performed because there was no confirmed candidemia positive case in our area during this study. Therefore, the current findings primarily reflect analytical performance in a controlled experimental model rather than confirmed clinical diagnostic performance.

The benchmarking strategy in this study utilized a conventional PCR assay based on the LAMP outer primers (F3/B3), allowing both methods to target the same *ITS2* region. This approach minimizes variability related to target selection and facilitates a controlled comparison of amplification performance. The use of LAMP outer primers in conventional PCR has been reported in previous studies as a baseline strategy for assay evaluation [19, 32]. Nevertheless, this configuration does not represent a standardized diagnostic method for candidemia. In clinical practice, validated molecular assays, including species‐specific PCR and qPCR, are generally preferred due to their higher reproducibility, sensitivity, and level of standardization [30, 31]. Accordingly, the comparative findings presented here should be interpreted within the context of this methodological limitation.

In addition, the evaluation of analytical specificity was performed using a limited panel of non‐albicans fungal species. Although no cross‐reactivity was observed, the panel does not fully represent the diversity of clinically relevant pathogens associated with bloodstream infections. Notably, important species such as *C*. *glabrata* [33, 34], members of the *C*. *parapsilosis* complex [33, 34], and the emerging multi–drug‐resistant pathogen *C*. *auris* [35] were not included. These organisms are frequently implicated in candidemia and are critical targets for differential diagnosis in clinical settings. Therefore, the specificity results should be interpreted with caution, as they may not fully reflect assay performance against all clinically relevant species. Future studies incorporating a broader range of fungal pathogens will be essential to more comprehensively evaluate assay specificity and support its clinical applicability.

Although the assay is described as a rapid detection method, it should be noted that the current workflow requires a DNA extraction step prior to amplification. Therefore, the approach represents an extraction‐based molecular assay rather than true direct amplification from whole blood. The total time‐to‐result, including DNA extraction and amplification, remains relatively short compared to conventional diagnostic workflows. However, the requirement for sample preprocessing may limit immediate point‐of‐care implementation. Future studies will focus on simplifying the workflow, including the potential development of extraction‐free or minimal‐processing protocols, to further enhance the applicability of the assay in clinical and resource‐limited settings.

Overall, the findings of this study indicate that the LAMP assay is a promising, rapid, and sensitive molecular approach for the detection of *C. albicans*. However, further validation using clinical specimens, expanded pathogen panels, and comparison with standardized molecular diagnostics will be necessary to establish its role in routine clinical practice.

## 5. Conclusion

In conclusion, the *ITS2*‐targeted LAMP assay represents a rapid and sensitive molecular approach for the detection of *C. albicans* under the experimental conditions evaluated in this study. By combining the species‐discriminatory capability of the *ITS2* region with the efficiency of isothermal amplification, the assay demonstrated the ability to detect low concentrations of fungal DNA and showed improved analytical sensitivity compared with conventional PCR. The assay also demonstrated consistent performance in artificially spiked blood samples, with a detection limit of 10^1^ CFU/mL across donor samples under controlled experimental conditions. In addition, the relatively simple workflow, short turnaround time, and minimal equipment requirements suggest potential applicability in laboratory settings with limited resources. However, the findings should be interpreted with caution due to several limitations, including the use of spiked samples rather than clinical specimens, the limited specificity panel, and the absence of comparison with standardized molecular methods such as qPCR. Therefore, further validation using clinical samples and expanded pathogen panels will be necessary to more comprehensively evaluate diagnostic performance and determine the potential applicability of the assay in clinical settings. Overall, the *ITS2*‐based LAMP assay shows promise as a complementary molecular method for the detection of *C. albicans* and warrants further investigation in future clinical studies.

## Author Contributions

Eakkapote Prompunt: conceptualization, methodology, validation, formal analysis, investigation, resources, data curation, writing–original draft, writing–review and editing, visualization, project administration, and funding acquisition. Sawitree Nangola: conceptualization, methodology, validation, formal analysis, investigation, resources, data curation, writing–original draft, writing–review and editing, visualization, supervision, and project administration. Kannika Jamnai, Nawapol Marome: methodology, validation, formal analysis, investigation, resources, and data curation. Nathaporn Phutthawong: methodology, validation, investigation, writing–original draft, and writing–review and editing. Thitima Sumphanapai, Parin Kamseng, Somphot Saoin, Chiraphat Kloypan, and Supaporn Khamchun: methodology, validation, formal analysis, investigation, data curation, and writing–review and editing.

## Funding

This research project was supported by the Thailand Science Research and Innovation Fund and the University of Phayao (Grant No. 258/2567) and by the Thailand Science Research and Innovation Fund, Fundamental Fund 2026 (Grant No. 2274).

## Conflicts of Interest

The authors declare no conflicts of interest.

## Data Availability

Data supporting the findings of this study are available from the corresponding author upon reasonable request.
